# Adherence to and usefulness of the national treatment guideline for urinary tract infections (UTI) in a risk area

**DOI:** 10.1186/s12875-022-01840-6

**Published:** 2022-09-07

**Authors:** A. A. van Driel, M. Mulder, E. E. Stobberingh, A. Verbon

**Affiliations:** 1 Department of Medical Microbiology and Infectious Diseases, Erasmus University Medical Center, ‘s-Gravendijkwal 230, 3015 CE Rotterdam, The Netherlands; 2 Department of Epidemiology, Erasmus Medical Center, ‘s-Gravendijkwal 230, 3015 CE Rotterdam, The Netherlands; 3 Department of Medical Microbiology, Maastricht University Medical Center, Maastricht, The Netherlands; 4 Department Infectious Disease Control, Municipal Public Health Service Rotterdam-Rijnmond (GGD Rotterdam), Rotterdam, The Netherlands

**Keywords:** Treatment guidelines, Empirical prescription, Antibiotic resistance, Uncomplicated urine tract infections, Community-acquired, Agriculture

## Abstract

**Background:**

To optimize antibiotic treatment and decrease antibiotic resistance, national treatment guidelines are available for urinary tract infections (UTIs) in general practice. The usefulness of these guidelines in risk areas for antimicrobial resistance such as cross border regions or areas with dense agriculture, is unknown.

**Methods:**

Midstream urine samples from women with symptoms of acute UTI visiting general practitioners (GPs) in the Westland area, a dense agriculture area, were microbiologically analysed, and patient characteristics, symptoms, previous and present antibiotic treatment were collected. The National Nivel data were used as reference for antibiotic resistance.

**Results:**

Of 310 women with symptoms of uncomplicated UTI, 247 (80%) had a culture proven *E. coli* UTI. Empirical antibiotic therapy was prescribed to 148 patients (48%) in total; in 7% of women with a negative and 52% with a positive urine culture. Having more than one symptom was associated with the prescription of antibiotics; travel history or previous antibiotic use for UTI were not. The isolated uropathogens were susceptible to the empiric antibiotic therapy in 98% of patients. Resistance to co-amoxiclav was higher (22%) than reported in the national data of 2004 (12%), 2009 (13%) and 2014 (9%), as was the prevalence of extended spectrum β-lactamase (ESBL): 3.4% in our study versus 0.1%, 1% and 2.2% in the national data respectively.

**Conclusion:**

The presence of environmental and socio-demographic risk factors for antibiotic resistance did not influence the empiric choice nor susceptibility for antibiotics advised by the national guidelines in women with uncomplicated UTI.

**Supplementary Information:**

The online version contains supplementary material available at 10.1186/s12875-022-01840-6.

## Background

Antimicrobial resistance (AMR) has been associated with the use of antimicrobial agents [1]. In humans, primary care physicians prescribe the majority of the consumed antibiotics (80%), mainly for the treatment of urinary tract infections (UTIs) and respiratory tract infections [2]. To optimize antibiotic use and prevent selection of antibiotic resistant bacteria, national guidelines for the treatment of common infections are available from the Dutch College of General Practitioners (NHG) [3]. An uncomplicated UTI is defined in women of 11 years and up, with no urological and nephrological problems, diabetes mellitus or using immunosuppression drugs. The empirical treatment of uncomplicated UTI according to the NHG guideline is based on patients symptoms without culture analyses and recommends nitrofurantoin as first choice and fosfomycin and trimethoprim as second and third choices. The antibiotic treatment options are based on national surveillance data. This guideline also describes other options for antibiotic prescription like telephone consulting when patients recognize symptoms of a previous UTI and a postponed prescription when patients agree to only start antibiotics when complaints don’t resolve. A urine culture is only sent in when empirical prescription is not effective or in patient with risk factors of a complicated UTI [3].

Antibiotics are not only prescribed in human health care, but also in livestock farming and in agriculture [4, 5]. Transmission of antibiotic resistant microbes from livestock to the environment and to humans has been reported [6]. In agriculture, several pesticides have been shown to affect the susceptibility of different bacteria for several antibiotics used in humans [7–9].

Indeed, studies report the occurrence of antibiotic-resistant (including extended spectrum β-lactamase (ESBL) producing) *E. coli* in irrigation water of vegetables for human consumption [10–12]. The effect of living in an area with a high risk of contamination of surface water with antibiotics or pesticides, such as a dense agricultural area, on the prevalence of AMR is still unknown [13]. Travel, especially from Asia harbours the risk of introducing AMR into the Netherlands [14, 15]. This risk may also exist in immigrants from South and East Europa as the prevalence of resistance in these countries is higher than in Northern countries, like the Netherlands [16].

Given the association between use of antibiotic drugs and AMR, the WHO advocates antimicrobial stewardship: to treat each patient with the most appropriate antimicrobial therapy, thereby minimalizing the risk of development of AMR [17]. The Dutch national guidelines support antimicrobial stewardship programs in general practitioners. Monitoring adherence to antibiotic guidelines in large databases has been shown to be a valuable antibiotic stewardship tool [18]. However, several studies report difficulties to adhere to the NHG guidelines [19]. One of the reasons was that the choice of the national empiric therapy was not applicable regionally due to differences in local resistance patterns. Ganzeboom et al. reported a low level of adherence to the guidelines (29%-50%) in high risk patients as the choice of the empiric therapy was not supported by bacteriological culture. They recommended that the empiric therapy for this group of patients should be based on the results of urine culture [20].

To explore the adherence and usefulness of the national guidelines for uncomplicated UTI, we investigated adherence to the guidelines for female patients with complaints of an uncomplicated UTIs in an area with sociodemographic (i.e. East European season immigrants) and environmental risk factors (i.e. presence of dense glasshouse agriculture) and determined whether the empiric therapy was in accordance with the culture results [3].

## Methods

### Patient recruitment and data collection

Patient recruitment took place at eight general practices in the Westland area of the Netherlands from March 2018 to June 2018. The Westland area is an area with dense agriculture, situated in the West of the Netherlands and characterized by many green glasshouses: 40% of the total surface of agriculture under glass (3.850.000.000 m2) is concentrated in this part of the country [21]. This region has also high numbers of (season)-immigrants from East Europe working in glasshouses, i.e. 75 persons per 10,000 inhabitants versus 7–15 per 10,000 inhabitants in other parts of the Netherlands [22].

General practitioners (GP) were asked to fill in a questionnaire upon presentation of women > 11 years with acute complaints of an uncomplicated UTI. Patient characteristics such as age and UTI symptoms such as pain/ burning, polyuria, urinary urgency, were collected as was information about, antibiotic therapy received for previous infections in the last 12 months and travelling abroad in the last 12 months. An uncomplicated UTI was defined as symptoms of pain or burning sensation, polyuria or urinary urgency without signs of tissue expansion like flank pain or fever. Furthermore, the actual empiric prescription was recorded. Exclusion criteria were pregnancy, fever (> 38 ℃), urological and nephrological problems, having a urinary catheter, diabetes mellitus and immunosuppression. Patients who received a prescription after laboratory culturing became available were not included. Also, patients with incomplete questionnaires were excluded. Included patients received care as usual for UTIs and the GPs did not have knowledge of the culture results at the moment of prescription or thereafter.

Informed consent was waived by local ethics committee (MEC-2017–1167).

### Isolation and Identification of uropathogens

Midstream urine samples were collected and sent the same day for microbiological analysis to the department of Medical Microbiology of the Erasmus Medical Centre Rotterdam, the Netherlands. Bacterial growth of the urine samples on blood agar and MacConkey (Oxoid) agarplates of ≥ 10^3^ cfu/ml was defined as an UTI [23]. Presence of three or more bacterial species was considered as contamination. For identification of the uropathogens, MALDI-TOF MS (Maldi MTB Compass 4.1, Bruker Daltonik GmbH, Bremen, Germany) was used. The antibiotic susceptibility was determined using disk diffusion (OXOID) or Vitek (Biomerieux) according to the EUCAST criteria 2017/2018 and supplemented with the E-test (Liofilchem) [24]. For confirmation of putative ESBL the Rosco ESBL Confirm Kit was used according to the Dutch Society of Medical Microbiology (NVMM) Guidelines of Laboratory detection of highly resistant microorganisms version 2.0 2017 [25].

The antibiotic resistance of the *E. coli* was compared to the resistance from studies performed in 2004, 2009 and 2014 among GP of the Sentinel Stations Network of NIVEL [26–29]. No data of more recent years are available after these years. The data from NIVEL are representative for age, gender, regional distribution and population density in The Netherlands [30].

### Statistical analysis

The X^2^ test and Fisher test were performed for comparing percentages of the categorical data (SPSS Inc. Chicago, IL, VS). A multivariate logistic regression was performed with as outcome variable the empirical prescription of an antimicrobial drug for this UTI episode and as variables age, number of symptoms, positive culture, UTI, antibiotic use and travelling abroad in the year before participating in this study. A *p-*value of < 0.05 was considered statistically significant.

## Results

### Patients characteristics

**Fig. 1:**
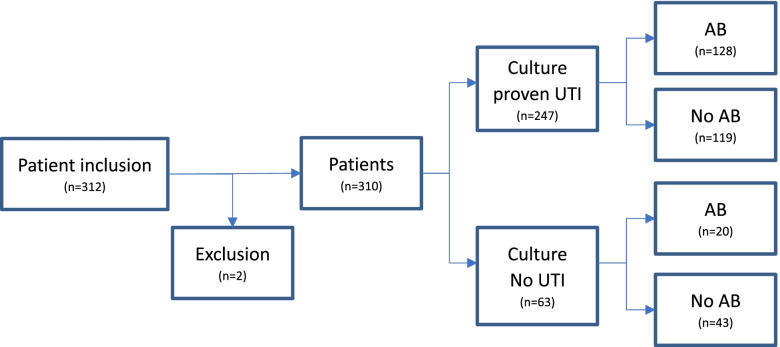
Inclusion of patients with symptoms of an UTI. Figure 1 shows the inclusion of patients presenting with UTI symptoms in 8 GP’s in the Westland and whether empirical antibiotics (AB) were prescribed. Two patients were excluded due to an incomplete questionnaire (*n* = 2). The diagnosis of UTI was confirmed by laboratory culture analyses after clinical presentation and diagnoses of the GP

During the study period, 312 women visited eight practices of general physicians with symptoms of an uncomplicated UTI. Due to incomplete questionnaires, two patients were excluded. Of the remaining 310 patients (median age 57 years, range 11–93 years), 247 (79.7%) had a culture proven UTI (Fig. 1). The most reported symptoms were pain/burning sensation (76.1%) and polyuria (50%). A prior history of UTI was reported by 174 (56.1%) patients and 183 (59%) were previously treated with nitrofurantoin (42.3%), fosfomycin (13.5%) or ciprofloxacin (6.8%). Travelling abroad over the last 12 months was reported by 17.1% of the patients (Table 1).

**Table 1:** Patients characteristics

	**Total (n, %)** **(*n* = 310)**	**UTI (n, %)** **(*n* = 247)**	**No UTI (n, %)** **(*n* = 63)**
**Age category (year)**			
11–20	23 (7.4)	18 (7.3)	5 (7.9)
21–50	100 (32.3)	77 (31.2)	23 (36.5)
51–70	115 (37.1)	96 (38.9)	19 (30.2)
≥ 71	72 (23.2)	56 (22.7)	16 (25.4)
**Symptoms**			
Pain/burning sensation	236 (76.1)	192 (77.7)	44 (69.9)
Polyuria	155 (50.0)	127 (51.4)	28 (44.4)
Urinary urgency	57 (18.4)	48 (19.4)	9 (14.3)
Haematuria	38 (12.3)	33 (13.4)	5 (7.9)
Flank pain	58 (18.7)	47 (19.0)	11 (17.5)
**UTI in previous year**	174 (56.1)	139 (56.3)	35 (55.6)
**AB in previous year**	183 (59.0)	146 (59.1)	37 (58.7)
Amoxicillin	18 (5.8)	12 (4.8)	6 (9.5)
Co-amoxiclav	6 (1.9)	4 (1.6)	2 (3.2)
Doxycycline	10 (3.2)	10 (4.0)	0 (0.0)
Nitrofurantoin	131 (42.3)	104 (42.0)	27 (42.9)
Fosfomycin	42 (13.5)	34 (13.8)	8 (12.7)
Trimethoprim	16 (5.2)	15 (6.1)	1 (1.6)
Ciprofloxacin	21 (6.8)	16 (6.5)	5 (7.9)
Norfloxacin	6 (1.9)	6 (2.4)	0 (0.0)
Co-trimoxazole	2 (0.6)	2 (0.8)	0 (0.0)
Clarithromycin	1 (0.3)	1 (0.4)	0 (0.0)
Flucloxacillin	4 (1.3)	3 (1.2)	1 (1.6)
Azithromycin	2 (0.6)	1 (0.4)	1 (1.6)
Cefuroxime	1 (0.3)	1 (0.4)	0 (0.0)
Metronidazole	1 (0.3)	0 (0.0)	1 (1.6)
**Traveling abroad**	53 (17.1)	45 (18.3)	8 (12.7)
Within Europe	37 (11.9)	31 (12.6)	6 (9.5)
Outside Europe	16 (5.2)	14 (5.7)	2 (3.2)
**Empirical AB**	148 (47.7)	128 (51.8)	20 (31.7)
Amoxicillin	1 (0.3)	1 (0.4)	0 (0.0)
Co-amoxiclav	1 (0.3)	0 (0.0)	1 (1.6)
Nitrofurantoin	112 (36.1)	97 (39.3)	15 (23.8)
Fosfomycin	19 (6.1)	16 (6.5)	3 (4.7)
Trimethoprim	9 (2.9)	8 (3.2)	1 (1.6)
Ciprofloxacin	5 (1.6)	5 (2.0)	0 (0.0)
Norfloxacin	1 (0.3)	1 (0.4)	0 (0.0)
none	162 (52.3)	119 (48.2)	43 (68.3)

Table 1 shows the patient characteristics of the study. The first column (total) shows the characteristics of all patients included in the study, Table 1 the second column (UTI) the characteristics of the patients with a culture confirmed UTI and the third column the characteristics of the patients without a culture confirmed UTI

### Empirical antibiotic therapy

Empirical antibiotic therapy was prescribed to 48% (*n* = 148) of the 310 patients, and the most frequently given antibiotics were nitrofurantoin in 112 patients (36%), fosfomycin in 19 patients (6%) and trimethoprim in nine patients (3%). Twenty of the 310 patients (7%) were empirically treated with an antibiotic although their culture could not confirm an UTI (Fig. 1). Of the 247 patients with a laboratory confirmed UTI, 119 (48.2%) received no empiric antibiotic therapy.

In the multivariate model, patients with two (OR 2.50; 95% CI 1.46 – 4.29) or three or more (OR 4.07; 95% CI 2.07 – 8.01) symptoms were found to be more often prescribed antibiotics than patients with no or one symptom. Furthermore, patients with a culture proven UTI afterwards were more often prescribed empirical antibiotic therapy (OR 1.98; 95% CI 1.06 – 3.69) than patients with a negative culture. Traveling abroad, either in or outside Europe, and previous use of antibiotics were not associated with prescription of antibiotics (Table 2).

**Table 2:** Factors associated with empirical antibiotic prescribing of patients visiting the GP for complaints of an UTI

	OR (95% CI)
	**Multivariate model**
**Age**	1.01 (0.99–1.02)
**Previous UTI (< 1 year)**	0.42 (0.12–1.51)
**Number of symptoms**	
No or 1 symptom	Ref
2 symptoms	2.50 (1.46–4.29)*
3 or more symptoms	4.07 (2.07- 8.01)*
**Culture proven UTI**	1.98 (1.06–3.69)*
**Previous antibiotic use (< 1 year)**	1.74 (0.48–6.24)
**Traveling abroad (< 1 year)**	
No	Ref
Yes, Europe	0.85 (0.41–1.78)
Yes, outside Europe	0.51 (0.16–1.57)

Table 2 shows the associated factors of empirical antibiotic prescription of patients visiting the GP with complaints of an UTI. Associated factors are age; the number of symptoms patients were suffering from; a culture proven UTI; antibiotic use in the year before this study and traveling abroad in the last 12 months. * Significant with *p* < 0.05

### Antibiotic resistance

Urinary samples (*n* = 247) with one or two uropathogens were included resulting in 293 uropathogens analyzed. *E. coli* was the most frequently isolated uropathogen (*n* = 167, 57%) followed by *Enterococcus faecalis* (*n* = 23, 8%), *Klebsiella spp.* (*n* = 18, 6%), *Streptococcus agalactiae (Group B streptococcus)* (*n* = 9, 3%) and *Proteus mirabilis* (*n* = 9, 3%). The remaining 67 (23%) uropathogens include among others Enterobacteriales and non-fermenters, see supplementary table 1.

**Fig. 2:**
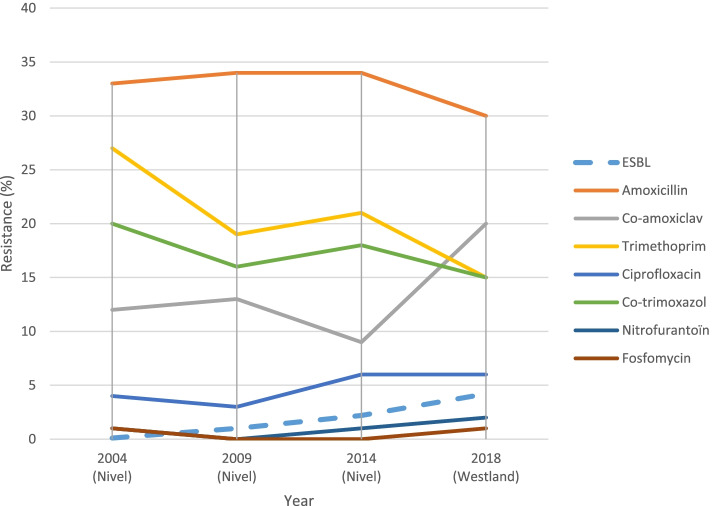
Percentages of antibiotic resistance of *E. coli* of the Westland (2018), NIVEL 2004, 2009, and 2014. Figure 2 shows the AMR percentage of the Westland in comparison to the NIVEL study of 2004, 2009 and 2014 [27–30]. Also the percentage of ESBL is shown

Antibiotic resistance was analysed for *E. coli* and compared to antibiotic resistance of three national studies from 2004, 2009 and 2014 (Fig. 2, supplementary table 2). Antibiotic resistance of the other uropathogens was not analysed due to low numbers. The highest percentage of antibiotic resistance of *E. coli* was found for amoxicillin (32%) followed by amoxicillin-clavulanic acid (22%), trimethoprim (15%) and co-trimoxazole (15%). The resistance rates to amoxicillin, trimethoprim and co-trimoxazole were in line with the national data. Resistance to amoxicillin-clavulanic acid was higher than in the national studies 12%, 13% and 9% in 2004, 2009 and 2014 respectively as was the prevalence of ESBL 3.4% versus 0.1%, 1% and 2.2% (Fig. 2). In nearly all cases (98%) the isolated uropathogen was susceptible to the empiric therapy.

## Discussion

In the present study we demonstrate the adherence and the usefulness of the NHG guidelines for the empiric treatment of UTI in female patients with complaints of an uncomplicated UTI in the Westland area. Only 7% of patients were not treated according to the guidelines and in 98% of patients the cultured uropathogen (*E. coli*) was susceptible to the empirically started therapy which was according to the NHG guidelines, i.e. nitrofurantoin as first choice and fosfomycin and trimethoprim as second and third choice. Traveling abroad or previous treatment for UTI were not associated with start of therapy (see Tables 1 and 2).

Our data indicate that in this risk area, GP’s adhere to the national guidelines and that the presence of environmental and socio-demographic risk factors did not influence the empiric choice nor the usefulness of the national guideline.

Risk factors for antibiotic resistance as described in the literature are recent and in the past antibiotic use, living in area with a high prevalence of resistance and travelling abroad [10–15].

Potential risk factors for a higher prevalence of resistance for Inhabitants of the Westland are exposure to pesticides due to a dense greenhouse agriculture and a high percentage of migrant workers [31–33], compared to people living in non-agricultural parts of the Netherlands [34]. Most migrant workers used to live in countries with a high antibiotic use and prevalence (East Europe) than in the Netherlands. Since the prevalence of antibiotic resistant *E. coli* in this study did not substantially differ from the national antibiotic resistance rates, the influence on antibiotic resistance of (season) immigrant workers seems negligible. We found only a higher resistance to amoxicillin-clavulanic acid, 22% in the Westland, versus 9% on national level in our study of 2014 [29]. Also national surveillance data showed an increasing trend of amoxicillin-clavulanic acid resistance from 2013 to 2018 was also reported; from 16 to 30% for patients ≥ 12 year [34]. This increase may partly be explained by higher minimum inhibitory concentration (MIC) breakpoints of the European Committee on Antimicrobial Susceptibility Testing (EUCAST) compared to the previously used Clinical and Laboratory Standards Institute (CLSI breakpoints) [34, 35].

According to the guideline amoxicillin-clavulanic acid is not recommended for uncomplicated UTI and was indeed not prescribed for this indication in the present study. The higher prevalence of resistance might be due to prescriptions for other indications such as respiratory tract infections [36]. Because of the lack of resistance data in women with uncomplicated UTI in 2018 we compared the present results with the latest national NIVEL data of 2014 [29]. In the last 10 years, from 2004- 2014 no upward trend was observed in antibiotic resistance of *E. coli* UTI. Use of other national data such as Nethmap [34] was not deemed useful because only urine culture after treatment failure of the GP patient were analysed, which may lead to higher levels of resistance.

An potential risk for antibiotic resistance in the Westland is the use of pesticides for agriculture [7–10]. None of the pesticides used as described in the annual report of the District Water Consult Board of Delfland is known to influence, in vitro nor in vivo (this study), the phenotypic antibiotic resistance of nitrofurantoin, fosfomycin, or co-trimoxazole.

Next to antibiotic use another risk factor for the introduction and spread of antibiotic resistance is travelling to regions with a higher prevalence of resistance compared to the Netherlands [15, 37, 38]. Travelling in or outside Europe was not related to AMR in our population, however only 18% of the women reported to have been travelling abroad. However, since in this study the prevalence of antibiotic resistant *E. coli* did not substantially differ from the national antibiotic resistance rates, the influence on antibiotic resistance of (season) immigrant workers seems negligible.

The increase in ESBL rates is recognized worldwide and many surveillance studies monitor the prevalence [39]. The occurrence of ESBL in this study was 3.4% compared to 2.2% at the national level in 2014 [29]. Our data are in line with the gradually upward trend in prevalence of ESBL percentage observed; 2004 of 0.1% to 1.0% in 2009 on a national level [26–29, 34].

We found an antibiotic match of 98% between the choice of the agent and the susceptibility of the isolated uropathogen, which support the correct empirical treatment of UTI by the participating GP’s and the adherence to the national NHG guideline of UTI. These results are in contrast to studies of Ganzeboom et al. and Lugtenberg et al. where regional differences of resistant patterns were found and no adherence to the NHG guideline [19, 20].

### Strengths and limitations

The strengths of the study is the careful control of the adherence and the usefulness of the NHG guidelines for the treatment of uncomplicated UTI in an area with socioeconomic and environmental risk factors. The adherence include not only the choice of the agent but also the decision when to start treatment. In our study 48% (*n* = 119) patients with a proved UTI were not empirically treated. The NHG guidelines describe the possibility of a postponed prescription including educating patients about the natural course of an UTI, (i.e. 20% of the UTI resolve spontaneously) and shared decision making. Adherence to national guidelines will contribute to control the antibiotic resistance problem. A limitation is the number of participating GP and patients included and the lack of follow -up data to determine the effectiveness of the policy prescribed. The low number of patients limit the applicability of our results for other areas in the Netherlands.

Here, we assessed whether national antibiotic guidelines are an useful antibiotic stewardship tool in general practice. Adherence of GP’s to these guidelines was high. Few patients were treated incorrectly (i.e. with a negative urine culture) and nearly all cultured bacteria were susceptible to the antibiotics given. The number of symptoms was associated with empirical antibiotic therapy, travel history and previous antibiotic use were not. When confirmed in larger studies, such factors may be included in guidelines for antibiotic therapy in women with uncomplicated UTI.

## Supplementary Information

**Additional file 1: Supplementary Table 1.** Percentages of uropathogens.** Supplementary Table 2.** Percentages of antibiotic resistance of *E.* *coli* in patients from the Westland area in comparison with data from studies of NIVEL 2004-2014 in the Netherlands

## Data Availability

The anonymized transcribed data from the current study are available from the corresponding author on reasonable request.
