# Supplementary material for: Adherence to and usefulness of the national treatment guideline for urinary tract infections (UTI) in a risk area
Source: BMC Prim Care. 2022 Sep 7;23:224. doi: 10.1186/s12875-022-01840-6 (PMC9450366; doi:10.1186/s12875-022-01840-6)
Supplement: Supplementary file 1 — Additional file 1: Supplementary Table 1. Percentages of uropathogens. Supplementary Table 2. Percentages of antibiotic resistance of E. coli in patients from the Westland area in comparison with data from studies of NIVEL 2004-2014 in the Netherlands [file 12875_2022_1840_MOESM1_ESM.docx]

**Supplementary Table 1: Percentages of uropathogens**

| **Pathogen** | **n (%)** |
| --- | --- |
| Escherichia coli | 203 (57.2) |
| Enterococcus faecalis | 23 (6.5) |
| Klebsiella species | 17 (4.8) |
| Streptococcus agalactiae (hemolytic streptococci group B) | 10 (2.8) |
| Proteus mirabilis | 9 (2.5) |
| Enterobacter species | 7 (2.0) |
| Acinetobacter species | 5 (1.4) |
| Staphylococcus saprophiticus | 4 (1.1) |
| Pseudomonas aeruginosa | 3 (0.8) |
| Staphylococcus aureus | 3 (0.8) |
| Citrobacter freundii | 2 (0.6) |
| Morganella morganii | 2 (0.6) |
| Raoultella ornithinolytica | 2 (0.6) |
| Other | 3 (0.8) |

Table 3 shows the percentages of the uropathogens that caused the urinary tract infection in this study. Please note that cultures with one or two uropathogens were defined as a culture proven urinary tract infection, , whereas cultures with three or more pathogens were considered as contamination and were excluded in this analysis.

**Supplementary Table 2. Percentages of antibiotic resistance of *E. coli* in patients from the Westland area in comparison with data from studies of NIVEL 2004-2014 in the Netherlands**

|  | Amoxicillin | Co-amoxiclav | Trimethoprim | Ciprofloxacin | Co-trimoxazole | Nitrofurantoin | Fosfomycin |
| --- | --- | --- | --- | --- | --- | --- | --- |
| NIVEL 2004  n = 2024  ESBL 0.1% | 33 | 12 | 27 | 4 | 20 | 1 | 1 |
| NIVEL 2009  n = 719  ESBL 1.0% | 34 | 13 | 19 | 3 | 16 | 0 | 0 |
| NIVEL 2014  n = 689  ESBL 2.2% | 34 | 9 | 21 | 6 | 18 | 1 | 0 |
| Westland 2018  n = 203  ESBL 3.4% | 32 | 22 | 15 | 7 | 15 | 2 | 1 |

Table 4 shows the percentage of antibiotic resistant *E. coli* including ESBL of the Westland study (2018) in comparison with the NIVEL study of 2004, 2009 and 2014 [27-30-28]. The NIVEL study is a national surveillance study performed of unselected *E. coli* isolated from GP’s of the Sentinel Stations network of NIVEL in The Netherlands [31]. The network is representative for age, gender, regional distribution and population density.
